# A Comparative Study on Multifactor Dimensionality Reduction Methods for Detecting Gene-Gene Interactions with the Survival Phenotype

**DOI:** 10.1155/2015/671859

**Published:** 2015-08-03

**Authors:** Seungyeoun Lee, Yongkang Kim, Min-Seok Kwon, Taesung Park

**Affiliations:** ^1^Department of Mathematics and Statistics, Sejong University, Seoul 143-747, Republic of Korea; ^2^Department of Statistics, Seoul National University, Seoul 151-747, Republic of Korea; ^3^Interdisciplinary Program in Bioinformatics, Seoul National University, Seoul 151-747, Republic of Korea

## Abstract

Genome-wide association studies (GWAS) have extensively analyzed single SNP effects on a wide variety of common and complex diseases and found many genetic variants associated with diseases. However, there is still a large portion of the genetic variants left unexplained. This missing heritability problem might be due to the analytical strategy that limits analyses to only single SNPs. One of possible approaches to the missing heritability problem is to consider identifying multi-SNP effects or gene-gene interactions. The multifactor dimensionality reduction method has been widely used to detect gene-gene interactions based on the constructive induction by classifying high-dimensional genotype combinations into one-dimensional variable with two attributes of high risk and low risk for the case-control study. Many modifications of MDR have been proposed and also extended to the survival phenotype. In this study, we propose several extensions of MDR for the survival phenotype and compare the proposed extensions with earlier MDR through comprehensive simulation studies.

## 1. Introduction

In early genome-wide association studies (GWAS), massive amounts of results have been reported on the associations between single-nucleotide polymorphisms (SNPs) and diseases. By now, 2,051 studies and 14, 836 causal variants (*p* value ≤5.0 × 10^−8^) have been added to catalogue of published Genome-Wide Association Studies [1]. However, it has been found that the effective sizes of the loci identified via GWAS are relatively small and a large proportion of heritability is still missing. This missing heritability problem has been studied by either considering gene-gene and gene-environment interactions or investigating rare variants based on new generation sequencing technology.

Traditional statistical methods are not well suited for detecting such interactions since the number of SNPs and their interactions increase exponentially. To address these issues, many bioinformatics methods for identifying gene-gene interactions have been proposed and one such method is multifactor dimensionality reduction (MDR) [2]. The MDR method is a computationally efficient method for detecting higher-order interactions between genes (and/or gene-environmental factors) and a binary phenotype. The key idea of MDR is to reduce multidimensional genotypes into one-dimensional binary attributes by using a well-defined classifier. Many modifications and extensions of MDR have been developed, which include log-linear models [3], generalized linear models [4], and model-based methods [5]. Among those, the generalized multifactor dimensionality reduction (GMDR) method extends MDR to both dichotomous and continuous phenotypes and allows for the adjustment of covariates such as age, sex, and other clinical variables.

In this study, we focus on gene-gene and/or gene-environment interactions associated with the survival phenotype. In a prospective cohort study, survival time has been one of the important phenotypes in studies of associations with gene expression levels measured by high-throughput microarray technology. Similarly, it has been important to identify the effect of SNPs on the survival phenotype in GWAS. A series of extensions of MDR to the survival phenotype has recently been proposed, which includes Surv-MDR [6], Cox-MDR [7], and AFT-MDR [8]. Those methods propose new statistics for classifying multilevel genotypes into a binary attribute under the MDR framework. However, as shown in the earlier simulation results [8], Cox-MDR has reasonable power in most cases and is robust to the censoring fraction, while AFT-MDR has similar power as Cox-MDR under no censoring but is very sensitive to the fraction of censoring. It is shown that the power of AFT-MDR substantially reduces, when the fraction of censoring increases more than 30%. That is why we propose two extensions of AFT-MDR, called dAFT-MDR and rAFT-MDR, to improve the power of AFT-MDR under heavier censoring.

Recently, a simple approach to MDR analysis of gene-gene interactions for quantitative traits, called QMDR, has been proposed [9]. The QMDR method replaces the balanced accuracy with a *t*-test statistic as a score to determine the best interaction model, which yields much less computing load. We extend the idea of quantitative MDR (QMDR) algorithm to Cox-MDR and AFT-MDR methods and propose two extensions of QMDR, called qCox-MDR and qAFT-MDR.

We compare the power of the proposed methods for various parameters including heritability, minor allele frequency (MAF), and censoring proportion with and without adjustment of covariates. It has been found that the improvements of AFT-MDR are less sensitive to censoring fraction than the original AFT-MDR but tend to have less power as the effect of covariate increases. On the other hand, the improvement of Cox-MDR is relatively robust to censoring fraction and tends to have reasonable power across many combinations of parameters.

## 2. Materials and Methods

### 2.1. Surv-MDR, Cox-MDR, and AFT-MDR

Since the MDR method has been originally proposed for a binary phenotype in case-control study, it was extended to quantitative traits and various sampling designs. Among those, the Surv-MDR was first proposed [6] for the survival phenotype by using the log-rank test statistic to classify the multi-genotypes into high and low risk groups. It replaces balanced accuracy by log-rank test statistics to determine the best model. However, Surv-MDR cannot allow for covariate adjustment, although adjustment of individual-specific covariates is very important in association studies to remove the confounding effect of covariates.

To overcome the drawback of Surv-MDR, the Cox-MDR method was proposed [7], in which the martingale residual of a Cox model is used as a new score for classifying high and low risk groups. In other words, if the sum of martingale residuals is positive for a specific genotype combination, then the corresponding genotype combination is classified as high risk group or low risk group, otherwise. Once all of genotype combinations are classified as either high or low risk group, the same procedure of original MDR algorithm is implemented to find the best interaction model. Since the martingale residual is obtained from a Cox model with adjusting covariates, the confounding effect of the covariates can be adjusted. It was shown from the simulation result in [7] that Cox-MDR has greater power than Surv-MDR and becomes much better when the effect of covariate increases. Furthermore, Cox-MDR keeps reasonable power even when the fraction of censoring increases, which implies that Cox-MDR is robust to heavier censoring.

Similarly, the AFT-MDR method has also been proposed by using the standardized residual as a new classifier under the accelerated failure time model [8] and the power of AFT-MDR is compared with that of Cox-MDR. As shown in the simulation results [8], the power of Cox-MDR seems to be reasonable in most cases and be robust to the fraction of censoring while the power of AFT-MDR decreases sensitively as the fraction of censoring increases, whereas it has similar power as Cox-MDR under no censoring. From the simulation results, it is shown that the power of AFT-MDR substantially reduces when the fraction of censoring increases more than 30%. Since censoring is very common to occur in survival data, we need to make AFT-MDR more robust to heavier censoring.

### 2.2. Improvements of AFT-MDR: dAFT-MDR and rAFT-MDR

As mentioned in the previous section, the improvement of AFT-MDR is needed to make it more robust to the fraction of censoring. Based on the simulated data in [8], the distribution of the standardized residual tends to have a long tail as the censoring fraction increases. Then the outliers may have a strong impact on the sum of the standardized residuals in AFT-MDR as those do on the mean value. We consider two different improvements to reduce the effect of the extreme values on the sum of standardized residuals in AFT-MDR.

We first transform the continuous standardized residual into a binary variable instead of taking their sum as done in AFT-MDR. In other words, the individual having the positive standardized residual is regarded as a control, whereas the individual having the negative standardized residual is regarded as a case. As a result, all data is discretized into 0 or 1 and then the original MDR algorithm is implemented, which is called dAFT-MDR (discretized AFT-MDR). Though dAFT-MDR is based on a binary value as the original MDR, it can adjust the covariate effect using the standardized residual of the AFT model, whereas the original MDR cannot adjust the covariate effect.

Secondly, we specify the lower and upper bounds of the standardized residuals and replace the extreme values of the standardized residuals beyond these bounds by either lower or upper bounds. Then we apply the algorithm of AFT-MDR, which is called rAFT-MDR (restricted AFT-MDR). By replacing the extreme values by the prespecified thresholds, the effect of the outliers on the standardized residual may be weakened when the distribution of the standardized residual is extremely skewed under the heavier censoring. However, the determination of threshold of the lower and upper bounds seems to be arbitrary and it should be considered with the behavior of the standardized residuals.

### 2.3. Improvements of Cox-MDR and AFT-MDR: qCox-MDR and qAFT-MDR

Recently, a simple MDR approach called QMDR for the quantitative trait has been proposed [9], in which the *t*-test statistic is used to determine the best interaction model in the frame of MDR. The key idea of QMDR can be easily adapted to modify Cox-MDR and AFT-MDR since both the martingale and standardized residuals are quantitative variables.

For Cox-MDR, we obtain the mean value of the martingale residual for each genotype combination and then compare it with the overall mean of the martingale residual. If the mean value of the martingale residual from the specific genotype combination is greater than the overall mean, the corresponding genotype is considered high risk group. Otherwise, it is considered low risk group, since the larger value of martingale residual has higher risk than expected. Once all of the genotypes are classified as high risk and low risk groups, a new binary attribute is created by pooling the high risk genotype combinations into one group and the low risk into another group. Then we use a *t*-test statistic to test the significant difference between high and low risk groups and choose the best model. The cross validation procedure for QMDR is the same as that used in original MDR. The difference is that the training score and testing score from the *t*-test statistics are used instead of training and testing balanced accuracies. As done in MDR, the training scores to determine the best *k*-order interaction model are computed and the maximum testing score is used to identify the best overall model. Similarly, the AFT-MDR method is also improved by using *t*-test statistic calculated from the standardized residuals of high and low risk groups. These improvements are called qCox-MDR and qAFT-MDR, respectively.

## 3. Simulation Results

We propose various improvements of AFT-MDR and Cox-MDR to increase the power for detecting gene-gene interactions with the survival phenotype. We implement the comprehensive simulation studies to compare the power of these improvements with those of original AFT-MDR and Cox-MDR.

For the simulation studies, the two disease-causal SNPs are considered among 20 unlinked diallelic loci with the assumption of Hardy-Weinberg equilibrium and linkage equilibrium. For the covariate adjustment, we consider only one covariate which is associated with the survival time but has no interactions with any SNPs. The simulation datasets are generated from different penetrance functions which define a probabilistic relationship between a status of high or low risk groups and SNPs. We consider eight different combinations of two minor allele frequencies of 0.2 and 0.4 and the four different heritabilities of 0.1, 0.2, 0.3, and 0.4. For each of the eight heritability-MAF combinations, a total of 5 models are generated, which yield 40 epistatic models with various penetrance functions, as described in [10].

Suppose that SNP1 and SNP2 are the two disease-causal SNPs and let  *f*_*ij*_  be an element from the  *i*th row and  *j*th column of a penetrance function. Then we have the following penetrance function:

(1) $$\begin{array}{c} f_{ij}=P\left(\;\text{high risk}\;|\;\text{SNP}1=i,\text{SNP}2=j\;\right). \end{array}$$

We generate 200 high risk patients and 200 low risk patients for each of the 40 models which depend on the penetrance function, MAF, and heritability. A more detailed description about the heritability assumption is given in [11]. For each dataset, we implement 5-fold cross validation and repeat it 10 times to reduce the fluctuation due to chance of divisions of the data. As a result, we have 100 datasets for each model.

To generate the survival time, we consider three different models: log-normal, Weibull, and Cox model. For each model, the effect size of the genetic factor is fixed as 1.0 and the effect sizes of adjusted covariate are given as *γ* = 0.0, 1.0. For the censoring fraction, we consider three different censoring proportions,  *C*_*p*_ = 0.0, 0.3, 0.5, because the power of AFT-MDR shows substantially decreasing trend when the censoring is heavier than 0.3 in the previous simulation results [8].

First, we check whether the false detection rate is close to the expected value when there is no gene-gene interaction effect because the best model is selected using the maximum balanced accuracy in the algorithm of MDR. To do this, we generate 100 datasets from each of the 40 models, which is a total of 4000 null datasets. Here the false detection rate is estimated as the percentage of times that the method randomly chooses the two disease-causal SNPs as the best model out of each set of 100 datasets for each model.  Table 1 shows the false detection rate for AFT-MDR, dAFT-MDR, rAFT-MDR, Cox-MDR, qCox-MDR, and qAFT-MDR for the log-normal distribution when the effect size of the adjusting covariate is given as *γ* = 0.0. Since only two disease-causal SNPs are considered among 20 SNPs, the expected false detection rate is given as 0.005. As shown in Table 1, the false detection rate varies from 0.002 to 0.008 across the combination of censoring proportion and MAF. For other simulation settings, the false detection rate behaves similarly as shown in Table 1 though not displayed here. It can be concluded that the false detection rate is close to the expected value.

For the power, we consider 100 simulated datasets for each of the 40 models, including two disease-causal SNPs, and we selected the best model over all possible two-way interaction models without and with adjustment of covariates, respectively. The power of dAFT-MDR is estimated as the percentage of times dAFT-MDR correctly chooses the two disease-causal SNPs as the best model out of each set of 100 datasets for each model. The power of the other improvements is defined as the same way of that of dAFT-MDR.

**Figure 1:**
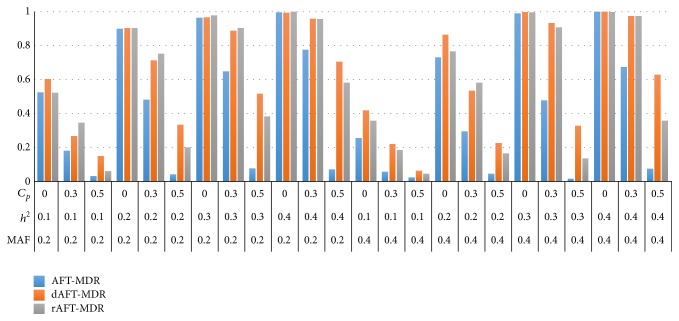
Comparison of the power of AFT-MDR, dAFT-MDR, and rAFT-MDR for the log-normal distribution when *γ* = 0.0. ^*^MAF: minor allele frequency; *h* ^2^: heritability; *C*_*p*_: censoring proportion.

**Figure 2:**
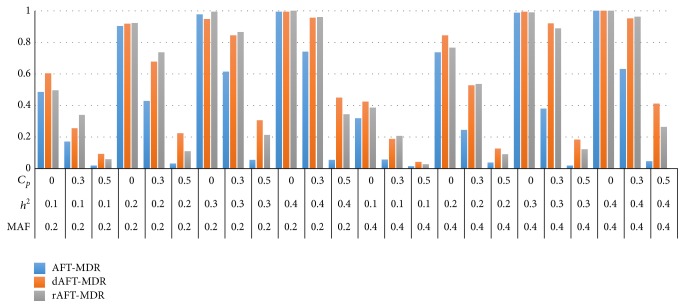
Comparison of the power of AFT-MDR, dAFT-MDR, and rAFT-MDR for the log-normal distribution when *γ* = 1.0. ^*^MAF: minor allele frequency; *h* ^2^: heritability; *C*_*p*_: censoring proportion.

Figures 1 and 2 present the power of AFT-MDR, dAFT-MDR, and rAFT-MDR under the log-normal distribution when *γ* = 0 and *γ* = 1, respectively. As shown in Figures 1 and 2, the power of AFT-MDR, dAFT-MDR, and rAFT-MDR has similar trend, which implies that the power of three methods increases as the heritability increases but is lower when the MAF increases from 0.2 to 0.4. As expected, the power of these three methods decreases as the censoring proportion increases from 0.0 to 0.5. In particular, the power of AFT-MDR decreases dramatically when the censoring proportion is lower than 0.3, whereas the power of dAFT-MDR and rAFT-MDR decreases gradually up to the censoring proportion of 0.3 but it decreases faster when the censoring proportion is 0.5. For example, when the MAF is 0.2, heritability is 0.2 and the censoring proportion increases from 0.0 to 0.3 and the power of AFT-MDR decreases from 0.9994 to 0.476 but the power of dAFT-MDR decreases from 0.9904 to 0.8068 and the power of rAFT-MDR decreases from 0.9992 to 0.8072, respectively. Furthermore, when the censoring proportion increases from 0.3 to 0.5, the power of AFT-MDR decreases to 0.0292, whereas the power of dAFT-MDR and rAFT-MDR decreases to 0.3322 and 0.1838, respectively. The degree of decreasing in power is substantially different by improvement in the sense that AFT-MDR hardly detects the significant gene-gene interactions associated with the survival time when the censoring is heavier than 0.5, whereas the improvements of AFT-MDR barely detect the gene-gene interactions. As the heritability increases, the power of AFT-MDR does not increase at all but the power of dAFT-MDR and rAFT-MDR increases up to 0.7026 and 0.5925, respectively. Comparing the power of dAFT-MDR with that of rAFT-MDR, these two improvements seem to behave similarly under the moderate censoring proportion but dAFT-MDR performs better than rAFT-MDR under the heavier censoring as mentioned. This implies that discretizing the standardized residual is more effective than restricting the extreme values as the censoring proportion is heavier than 0.5.

On the other hand, the power of AFT-MDR, dAFT-MDR, and rAFT-MDR behaves similarly when the effect of the covariate increases from *γ* = 0.0 to *γ* = 1.0 as shown in Figures 1 and 2. This is because the effect of covariate is adjusted by calculating the standardized residual from the AFT model with the adjusted covariates. In addition, the simulation results for the Weibull distribution show the same trend as those for the log-normal distribution though not shown here.

**Figure 3:**
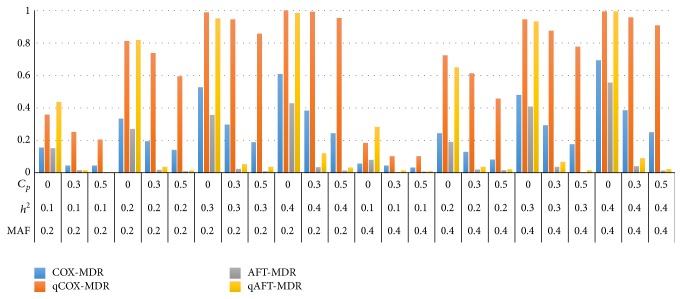
Comparison of the power of Cox-MDR, qCox-MDR, AFT-MDR, and qAFT-MDR for a Cox model when *γ* = 0.0. ^*^MAF: minor allele frequency; *h* ^2^: heritability; *C*_*p*_: censoring proportion.

**Figure 4:**
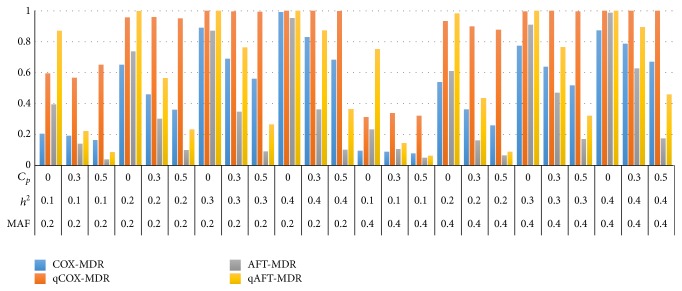
Comparison of the power of Cox-MDR, qCox-MDR, AFT-MDR, and qAFT-MDR for a log-normal distribution when *γ* = 0.0. ^*^MAF: minor allele frequency; *h* ^2^: heritability; *C*_*p*_: censoring proportion.

Figures 3 and 4 show the power of Cox-MDR, qCox-MDR, AFT-MDR, and qAFT-MDR for a Cox model and the log-normal distribution, respectively, when the effect size of the adjusted covariate is *γ* = 0.0. The power of these four methods performs similarly when the covariate effect is *γ* = 1.0. In addition, the power of these four methods for the log-normal distribution is almost the same as that for Weibull distribution though not shown here.

Comparing the simulation results shown in Figures 3 and 4, the power of Cox-MDR, qCox-MDR, AFT-MDR, and qAFT-MDR for a Cox model is rather lower than that for the log-normal model though these two power trends are consistent under the various combinations of the MAF, heritability, and the censoring proportion. The power of these four methods commonly increases as the heritability increases but decreases as the censoring proportion increases and the MAF increases from 0.2 to 0.4. However, the power of Cox-MDR and AFT-MDR is always lower than that of qCox-MDR and qAFT-MDR and decreases substantially as the censoring is heavier than 0.3. For a Cox model, when the MAF is 0.2, the heritability is 0.2 and the censoring proportion increases from 0.0 to 0.3, the power of Cox-MDR decreases from 0.334 to 0.196, and the power of AFT-MDR decreases from 0.270 to 0.018, respectively, whereas the power of qCox-MDR decreases from 0.812 to 0.738 and the power of qAFT-MDR decreases from 0.818 to 0.038, respectively. Furthermore, when the censoring is heavier than 0.5, the power of Cox-MDR and AFT-MDR decreases to 0.142 and 0.010, respectively, whereas the power of qCox-MDR and qAFT-MDR decreases to 0.594 and 0.014, respectively. As shown in Figure 3, only the power of qCox-MDR is robust to heavy censoring mechanism, whereas the power of Cox-MDR, AFT-MDR, and qAFT-MDR is very low when the censoring proportion is heavier than 0.3.

On the other hand, for a log-normal model, the power of Cox-MDR decreases from 0.650 to 0.458 as the censoring fraction increases to 0.3 when the MAF is 0.2 and the heritability is 0.2, whereas the power of qCox-MDR changes from 0.958 to 0.960. In addition, the power of Cox-MDR decreases to 0.360 as the censoring fraction increases to 0.5, but the power of qCox-MDR is 0.95, which implies that qCox-MDR is very robust to the censoring fraction. Under the same setting, however, the power of AFT-MDR decreases from 0.738 to 0.302 and the power of qAFT-MDR decreases from 0.998 to 0.564, respectively, as the censoring fraction increases to 0.3. As the censoring fraction increases to 0.5, the power of AFT-MDR and qAFT-MDR decreases to 0.098 and 0.232, respectively. This result is consistent for both the Cox model and the log-normal model, which implies that only the power of qCox-MDR is robust to heavy censoring, though the power of qAFT-MDR is rather higher for the log-normal model than that for Cox model. These trends are similar for Weibull distribution.

In summary, the simulation results show that AFT-MDR, dAFT-MDR, rAFT-MDR, and qAFT-MDR are more sensitive to heavy censoring (more than 0.5) than Cox-MDR and qCox-MDR across various situations. However, for the moderate censoring (less than 0.3), dAFT-MDR, rAFT-MDR, and qAFT-MDR perform much better than the original AFT-MDR.

## 4. Conclusions

Since many findings from GWAS have been published for the last decades, there is still a missing heritability problem. In order to search the missing heritability, we focus on gene-gene interactions because most of common diseases may be due to the complexity of gene-gene and/or gene-environment interactions rather than a single gene effect. Many plausible approaches have been developed by extending existing methods into a more general framework.

In this paper, we propose various improvements to AFT-MDR and Cox-MDR, which include dAFT-MDR, rAFT-MDR, qAFT-MDR, and qCox-MDR. The motivation to propose dAFT-MDR and rAFT-MDR is to improve the power of AFT-MDR because the performance of AFT-MDR is poor when censoring becomes heavier than 0.3. To reduce the effect of heavy censored observation, we discretize the standardized residual into a binary value, which yields dAFT-MDR. Alternatively, we truncate the extreme values and replace them by specified lower and upper bounds, which leads to rAFT-MDR. As shown in the simulation results, both AFT-MDR and rAFT-MDR have larger powers than the original AFT-MDR for the moderate censoring but still have low powers for the heavy censoring.

In addition, we considered the improvement of QMDR, which has been recently proposed in [9]. By regarding the martingale residual and the standardized residual as the quantitative traits, we adapted the main idea of QMDR and applied it to Cox-MDR and AFT-MDR, which yield qCox-MDR and qAFT-MDR, respectively. As shown in the simulation results, qCox-MDR and qAFT-MDR provided improved performances compared to those of the original Cox-MDR and AFT-MDR, respectively. In particular, qCox-MDR showed the consistent power regardless of the censoring fraction. However, qAFT-MDR yielded the weak power when the censoring fraction is heavier than 0.3. The censoring fraction seems to have a larger effect on the standardized residual than on the martingale residual. It would be desirable to consider how to make the standardized residual more robust to censoring mechanism.

In conclusion, the improvement of Cox-MDR, say qCox-MDR, has reasonable power and is robust to the heavy censoring, whereas the several improvements of AFT-MDR, say dAFT-MDR, rAFT-MDR, and qAFT-MDR, perform better than AFT-MDR but are not robust to heavy censoring. More studies on the behavior of the standardized residuals are needed to improve the power of AFT-MDR under the heavier censoring.

## Figures and Tables

**Table 1:** The false detection rate of AFT-MDR, dAFT-MDR, rAFT-MDR, Cox-MDR, qCox-MDR, and qAFT-MDR for the log-normal distribution with *C*_*p*_ and MAF when *γ* = 0.

MAF	*C*_*p*_	AFT-MDR	dAFT-MDR	rAFT-MDR	Cox-MDR	qCox-MDR	qAFT-MDR
0.2	0	0.008	0.004	0.006	0.006	0.008	0.003
0.2	0.3	0.002	0.005	0.008	0.006	0.007	0.005
0.2	0.5	0.007	0.005	0.004	0.005	0.004	0.003
0.4	0	0.003	0.006	0.006	0.004	0.008	0.006
0.4	0.3	0.004	0.004	0.005	0.003	0.006	0.003
0.4	0.5	0.007	0.006	0.005	0.006	0.005	0.008

MAF: minor allele frequency; *C*_*p*_: censoring proportion.
